# Variability of zinc, copper and lead contents in sludge of the municipal stormwater treatment plant

**DOI:** 10.1007/s11356-017-9338-1

**Published:** 2017-06-06

**Authors:** Tomasz Zubala, Magdalena Patro, Patrycja Boguta

**Affiliations:** 1 Department of Environmental Engineering and Geodesy, University of Life Sciences in Lublin, Leszczyńskiego 7, 20-069 Lublin, Poland; 2 Institute of Agrophysics, Polish Academy of Sciences, Doświadczalna 4, 20-290 Lublin, Poland

**Keywords:** Rainwater sewage, Sludge, Pollutants, Heavy metals

## Abstract

Several years of observations enabled us to assess the extent and variability of heavy metal contamination (Zn, Cu and Pb) of the sludge of the municipal stormwater treatment plant (the town of Puławy, Poland). In terms of size (high capacity) and innovation, it is the only facility of this kind in the country. It collects rainwater run-offs from two catchments (separate inlets) with a total area of about 500 ha. The concentration of the analysed metals is characterised by a large spatial and temporal diversity. The reason for this may be differences in the manner and intensity of use of drained surfaces and different hydraulic conditions (of sedimentation) prevailing in the particular treatment devices. The highest pollution was found in sediments in the grit chamber and in the part of the settler from the side of the interceptor supplying sewage, i.e. from the main traffic route of the town (heavy traffic and developed technical infrastructure). The best-quality sludge was retained in the pond for treated wastewater. In the pool of analysed components, the largest share is Zn, which amounts to about 85%. The content of heavy metals limits the possibility of the natural use of sludge from the municipal stormwater treatment plant. In chemical terms, they should be seen as a potentially dangerous waste and undergo remediation.

## Introduction

Rain sewage is characterised by high heterogeneity and quantitative and qualitative variability. It can be loaded with a large load of pollution and pose a significant threat to the environment if it runs off from heavily urbanised and industrialised areas (Barałkiewicz et al. 2014; Eriksson et al. 2005; Shirasuna et al. 2006; Zubala 2013). One of the most important components of rainwater sewage is a suspension, to which other contaminants are adsorbed, e.g. petroleum compounds or heavy metals (Garbarczyk and Gwoździej-Mazur 2005; Gromaire-Mertz et al. 1999; Sawicka-Siarkiewicz 2003; Wakida et al. 2014). Rainwater run-offs from urban catchments may contain up to 4000 mg dm^−3^ of suspensions, 6 mg dm^−3^ of zinc, 0.8 mg dm^−3^ of copper and 2 mg dm^−3^ of lead (Królikowski et al. 2006). According to Dąbrowski (2001), in urban area, rainwater is able to collect about 0.4 to 2.3 Mg of suspension from 1 ha of sealed surface. Due to the strong affinity of suspensions and metals, the special attention to the process of separation and safe disposal of sewage sludge should be paid. Heavy metal compounds are characterised by the high durability and the ability to accumulate in the environment, even if they are removed periodically and in small quantities (Giri and Singh 2015; Kabata-Pendias and Mukherjee 2007; Rötting et al. 2014).

Today, among the research aims, the assessment of the possibility of sustainable rainwater management (Arora and Reddy 2015; Tao et al. 2014; Yu et al. 2013), including their effective treatment (Chang et al. 2012; Nolde 2007; Read et al. 2008), is propounded on the front burner. The problem of the quality of sewage sludge produced in the process of sedimentation of suspensions and its potential impact on the environment is often put aside. The chemical properties of the deposit, including the degree of accumulation of heavy metals, are not sufficiently recognised. As a result, the removal of sediments is an important operating issue for municipal services. The flawed handling of them, as the storage in random and unsecured places, is observed frequently. There is a great need to identify precisely the potential threats from sediments to the ecosystems and individual components of the environment.

The most popular among solutions in the field of stormwater management (regarding practical and research activities) are currently small objects, usually localised in a spot, in the immediate vicinity of the drained surface, e.g. roads, car parks and petrol stations. These include separators, settlers, infiltration basins and retention-infiltration reservoirs (Langeveld et al. 2012; Moore and Hunt 2012; Tran and Kang 2013; Zubala and Patro 2015). The multiple-element systems of several hectares, receiving rainwater sewage from large areas and performing several functions at the same time, are—unfortunately—rarely used. This is due to economic and spatial constraints, or the lack of adequate knowledge and experience. Only few scientific studies include an assessment of their technical-operational parameters or operational efficiency, including the type and the amount of retained pollutants.

The aim of this article is to assess the variability of the content of zinc (Zn), copper (Cu) and lead (Pb) and the degree of pollution of sediments from the municipal stormwater treatment plant by these metals. The stormwater treatment plant collects the outflows of about 500 ha of Puławy town and is the only facility of this kind in Poland (extremely high capacity and innovation of used technology). The large size of the sewage treatment, the series-parallel locating of the devices, the variable hydraulic conditions and their associated periods of keeping of sewage (process of sedimentation) indicate the possible existence of a high variation of concentration of heavy metals in sediment in the subsequent purification steps.

## Material and methods

### The study area

The town of Puławy is located in southeastern Poland, on the right bank of the Vistula River, on the border of Mazovia Lowland and Lublin Upland. This area is characterised by a large diversity of geomorphological and geological forms (slopes and loess ravines, sandy and loamy glacial moraines, limestone outcrops, inland dunes) (Kondracki 2000). The average total annual precipitation in the study area is approximately 570 mm. During the year, 165 days with precipitation, with maximum heights in July, are recorded. The time of snow cover is 60 days. The average annual temperature of air reaches 8 °C (Kaszewski 2008).

The Puławy municipality occupies an area of 50.6 km^2^ and has a population of almost 50,000 inhabitants. About 97.6% of the population uses the water supply system, and 95.6%—the sewage system (exploitation of underground water intakes and mechanical-biological treatment plant). National road no. 12 and voivodeship road nos. 801 and 824 run through the town. In the area of Puławy, about 330 economic entities in the industrial sector and 460 in the construction sector are registered (US 2014).

**Fig. 1:**
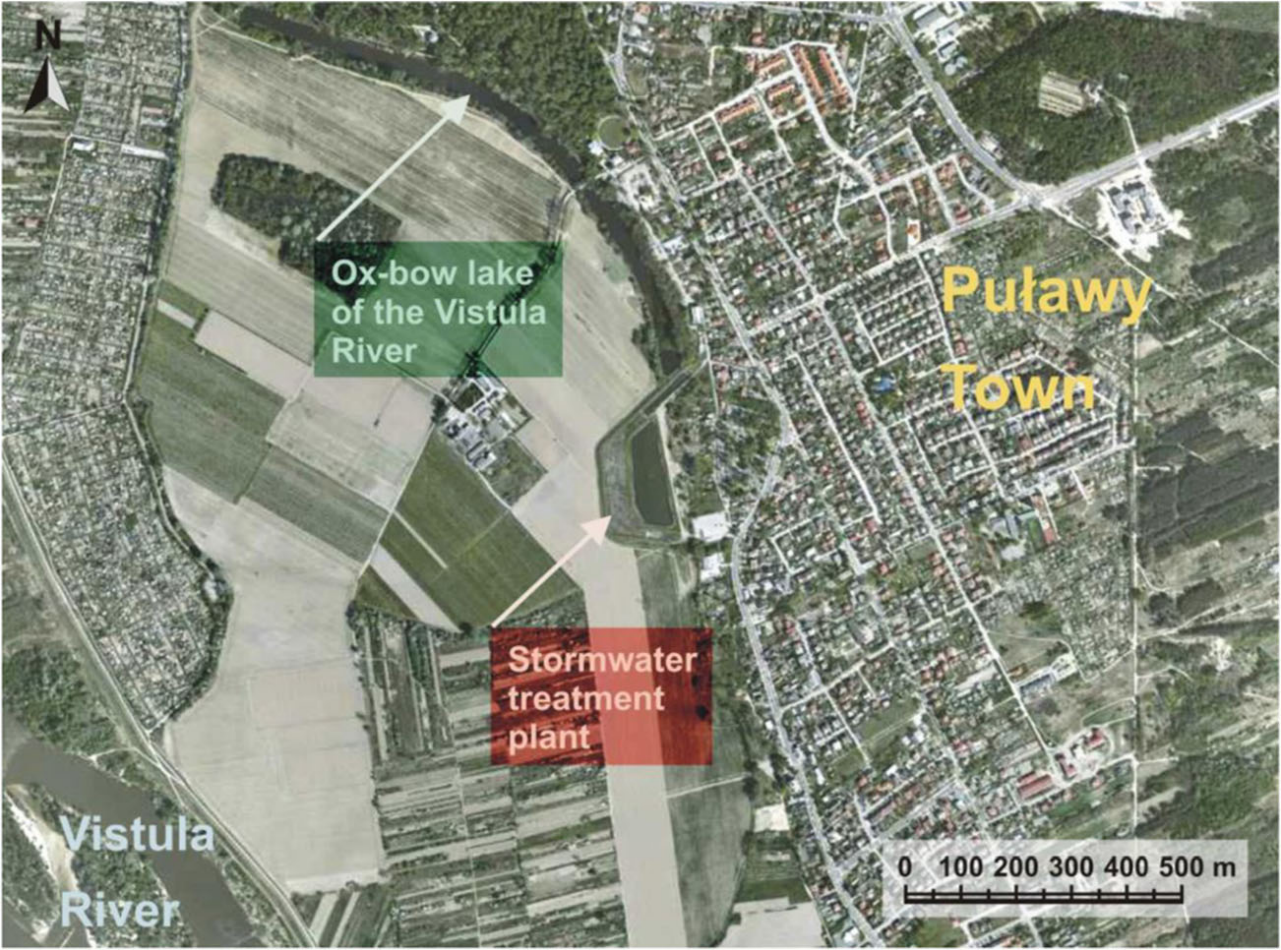
Location of the studied stormwater treatment plant (www.geoportal.gov.pl)

Since 1995, the stormwater treatment plant has functioned in the town. It is located on the lower flood terrace of the Vistula River in the vicinity of the oxbow lake (Fig. 1). Nearby, there are also large areas of arable land, a poorly developed area with old buildings and housing estate of family houses, and a Roman Catholic cemetery.

### Stormwater treatment plant

**Fig. 2:**
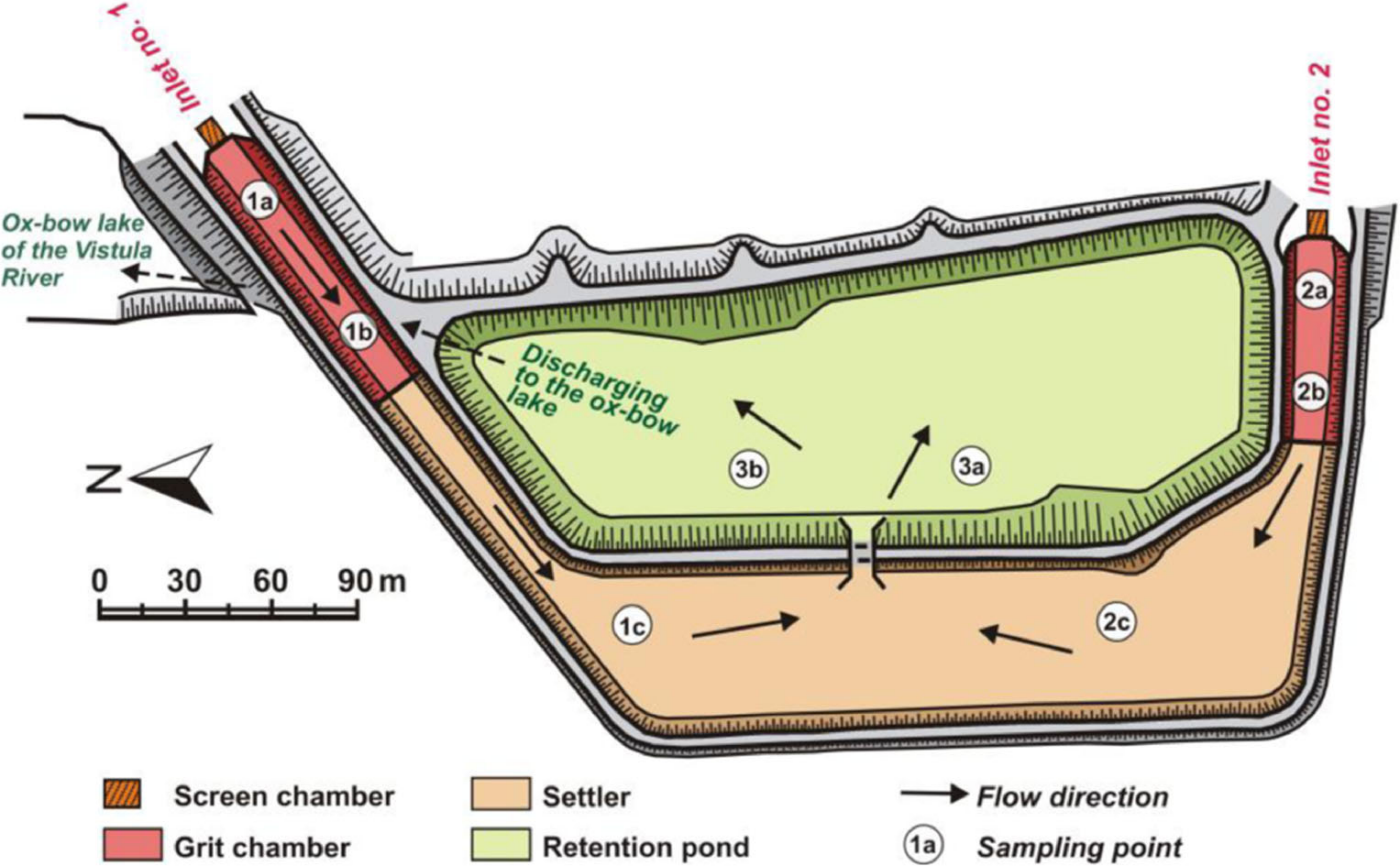
Scheme of the stormwater treatment plant with location of sampling points of bottom sediments

The most important elements of presented object are bars, grit chambers, settler and retention pond (Fig. 2). Transport of rainwater sewage uses only gravity. This is possible because of preserving the appropriate longitudinal slopes and differences in bottom elevation of the individual devices. During the flow, processes of self-cleaning occur—mainly straining, filtration, sedimentation, sorption, mixing, dilution, aeration and biological reactions. These types of phenomena are common in aquatic ecosystems and wetlands (Braskerud 2001; Dhote and Dixit 2007; Herrmann 2012; Zubala 2009). Treated sewage is discharged to the oxbow lake of the Vistula (the final receiver).

The urban stormwater drainage system consists of two main interceptors (diameter 1.4 and 1.6 m) and smaller side channels with utilities. Interceptor 1 receives sewage from an area of nearly 170 ha, and interceptor 2—of 300 ha. The sewage system drains the most urbanised areas of the town. Its range includes the main street of Puławy, a few residential districts (predominance of multi-family buildings) and areas of services, education and sports.

**Fig. 3:**
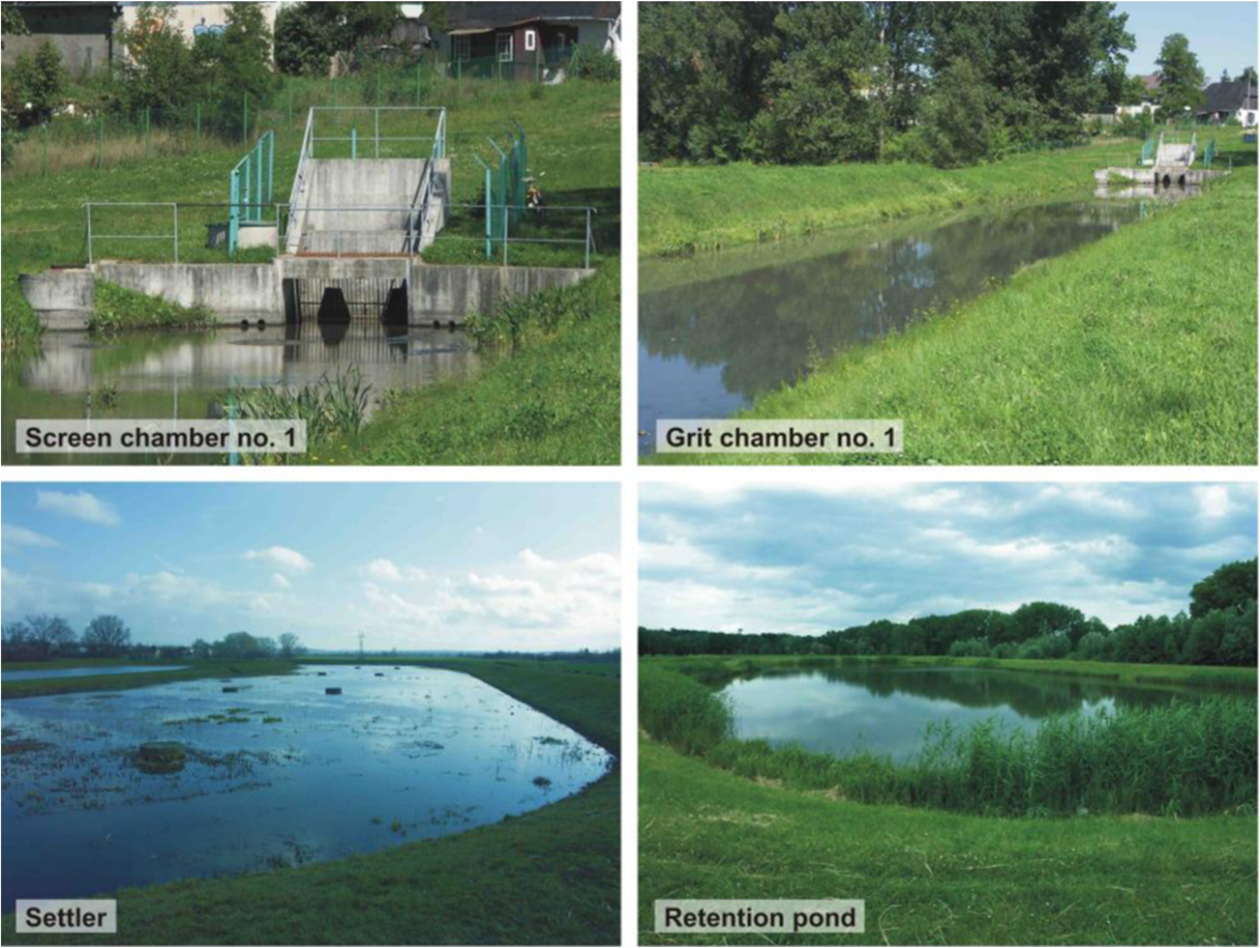
Basic devices of the stormwater treatment plant

In the inlet chambers of the studied stormwater treatment plant, flat bar screens with a clearance of 8 cm were installed and manually cleaned (separately for the two main interceptors) (Fig. 3). After the process of straining and stopping the larger solid contaminants, wastewater flows to grit chambers of 100 and 70 m length. The width of the bottom of these chambers is the same, 10 m, with an average active depth of 1 m. Designed speeds of liquid flow are 0.42 m s^−1^ (grit chamber 1) and 0.23 m s^−1^ (grit chamber 2). Parameters of the devices allow sedimentation of heavy suspended solids. The grit chambers are separated from the settler by overflows equipped with weirs. In the settler, wastewater flows into it from opposite directions and mixes. The length of the main part of the object is 190 m, and the width 46 m. The usable capacity reaches 12,540 m^3^, with a working depth of 1.2 m. After stopping light organic and mineral suspensions (slow flow), wastewater flows to the retention pond—the last facility of the sewage treatment plant (Fig. 3) through the culvert with weirs. The area of the reservoir is about 1.5 ha. The total capacity equals 38,100 m^3^, with the lower layer kept permanently, powered by groundwater (18,700 m^3^). The upper retention layer is discharged to the oxbow lake of the Vistula. Culverts with weirs are used for this purpose (two channels under the bottom of grit chamber 1).

The bottoms of the grit chambers and the settler are protected by openwork reinforced concrete slabs, laid on a geotextile. These objects are provided with drainage, which allows draining them rapidly, and then cleaning of the accumulated sludge. Another task of the drainage is to capture potential percolation, which reduces the possibility of contaminating groundwater. The retention pond is a typical earth structure. Like other devices, it is not sealed. It does not have its own drainage and technical reinforcements. From the farmlands, the stormwater treatment plant is surrounded by a band-shaped trench to prevent swamping the adjacent land. In the settler and at the base of dikes of the grit chambers and the retention reservoir, water-loving plants appear spontaneously. It is periodically mowed and removed from the area of the stormwater treatment plant.

### Sediment sampling

Sediment samples for laboratory analyses were taken in autumn of 2004, 2007, 2009 and 2015 using a sediment core sampler, a Beeker type from Eijkelkamp. Sampling of material with the intact structure was taken from the bottom of particular stormwater treatment facilities from the 0–10-cm layer. It took place the year after the removal of sludge from the grit chambers and the settler, as part of routine operational activities (sampling was preceded by accumulation for four seasons). During the study period, the user did not do any cleaning of the bottom of the retention pond due to a small amount of retained suspensions there.

The exact location of points of sediment sampling (Fig. 2):

- Grit chambers—15 m from the bar screen (samples 1a and 2a) and 3/4 of their length—75 m (1b) and 55 m (2b)
- Settler—100 m behind a baffle separating the grit chamber from the settler (half the distance between the baffle and the culvert of the retention reservoir)—samples 1c and 2c
- Retention pond—30 m from the culvert connecting the reservoir to the settler—samples 3a and 3b

### Methods of analysis

Before the start of laboratory analyses, samples were sieved and dried at 105 °C. About 2 g of each sample was weighed and ashed in a muffle furnace at 550 °C for 6 h. The ash was digested in 10 ml of aqua regia, then the solutions were filtered and deionised water was added to obtain a 50 cm^3^ final volume. The content of Zn, Cu and Pb was determined by atomic absorption spectrometry (AAS, contrAA 300, Analytik, Jena). The analyses were replicated three times, and the obtained data was averaged.

To assess the degree of contamination of sediments, the extreme and mean values of the analysed indicators for each checkpoint and each device were determined (Fig. 2). The percentage differences in metal concentrations between chosen positions were evaluated. The statistical variability of the results was determined based on the standard deviation and coefficient of variation.

## Research results and discussion

The concentrations of heavy metals (Zn, Cu and Pb) in the analysed sludge are characterised by a high spatial and temporal variation. This may be associated with the variable quality of influent rainwater (the method of land use, the type of suspension, weather conditions, etc.) and the used treatment technology (hydraulic conditions, time of keeping of sewage, operational treatments, etc.). Some regularity can be noticed when comparing deposits from particular facilities of the wastewater treatment. In each measurement period, the highest average concentrations of metals were found in the suspension accumulated in grit chamber 1 (inflow of sewage, i.e. from the main traffic route of the town), and the lowest in the retention pond (the reservoir of treated sewage) (Table 1). Sediments in grit chamber 2 were significantly less polluted than sediments in grit chamber 1 (Table 2), and only slightly more than in the pond (much lower intensity of economic use of the catchment of interceptor 2). The average metal contents in the settler were always between the averages in grit chambers 1 and 2 (preliminary treated sewage in the grit chamber mixes in the settler). With the exception of two cases (concentrations of Zn in the settler and the retention pond), arithmetic means and medians were similar, which shows a high homogeneity of the obtained results in the particular devices and the measurement dates (Table 1). The highest coefficients of variation characterised the concentration of metals in sludge from the settler. Also, in this device, the maximum values of studied pollutants were indicated in single dates (Zn—334.0 mg kg^−1^, Cu—31.9 mg kg^−1^, Pb—59.3 mg kg^−1^).

**Table 1:** Characteristic values of the analysed pollution indicators of sewage sludge

Variables	Stage	Minimal value	Maximum value	Average	Median	Standard deviation	Variation coefficient
Zn (mg kg^−1^)	Grit chamber no. 1	218.9	293.3	248.4	239.0	26.7	10.8
	Grit chamber no. 2	100.7	131.0	112.0	111.2	10.1	9.0
	Settler	54.2	334.0	156.3	135.8	90.7	58.0
	Retention pond	49.0	174.5	94.2	76.4	47.7	50.6
Cu (mg kg^−1^)	Grit chamber no. 1	15.0	30.4	19.7	18.0	5.3	27.1
	Grit chamber no. 2	6.0	10.4	8.6	8.7	1.4	16.8
	Settler	4.1	31.9	11.0	7.9	8.9	80.6
	Retention pond	4.7	11.6	6.7	6.2	2.1	31.9
Pb (mg kg^−1^)	Grit chamber no. 1	12.4	54.6	30.4	26.7	14.8	48.7
	Grit chamber no. 2	11.4	18.3	14.2	14.0	2.3	16.4
	Settler	5.6	59.3	21.0	17.1	16.6	79.2
	Retention pond	4.1	19.1	10.1	10.3	5.4	53.4

**Table 2:** Percentage differences between the average concentrations of metals at particular observation posts (“−” - decrease, “+” - increase)

Variables	Sampling points (comparison)					
	1a-2a	1a-1b	1b-1c	2a-2b	2b-2c	Settler-pond
Zn	−51.3	+11.0	−19.3	−4.8	−6.9	−39.7
Cu	−52.4	+8.5	−26.8	−8.9	−14.6	−39.1
Pb	−51.6	+14.8	−9.8	+6.6	−13.7	−51.9

Significantly higher concentrations of the studied metals in sediment samples taken at point 1a (the beginning of grit chamber 1) in comparison with samples from point 2a (the beginning of grit chamber 2) indicate the existence of a relationship between the method of land management of the catchment and the quality of outflowing rainwater. This connection is also confirmed by literature data (Chang et al. 2004; Goonetilleke et al. 2005; Petrucci et al. 2014). In the case of the object in Puławy, in the catchment of interceptor 1, there are more pollution sources than in the catchment of interceptor 2. In the first zone, there is a larger share of roads (including the main street of the town) with the diverse technical infrastructure and a significantly higher amount of traffic. Numerous studies have shown that the traffic routes are, for example, providers of large quantities of the suspensions deriving from corrosion and abrasion of vehicle components and road surfaces (Dąbrowski 2004; Kayhanian et al. 2003; Królikowski et al. 2006; Mangani et al. 2005). Worse quality of sludge near the inlet of grit chamber 1 was due to a near 50% increase in average content of Zn, Cu and Pb than in the sediment from the analogous parts of grit chamber 2 (Table 2). This concerned all years of the observation. Within the particular grit chamber, differences in the mean values of the analysed indicators were also found. Comparing points a and b, an increase in the concentration of metals in sediments of grit chamber 1 with distance from the bar screen can be seen. However, in sediments of grit chamber 2, similar trends were observed only in the case of Pb. These phenomena are likely the result of different hydraulic conditions prevailing in the particular zones of the grit chambers and related to distinct sedimentation of various fractions of the suspension. According to the design assumptions, wastewater in grit chamber 1 flows twice as fast as in grit chamber 2, and the speed in both devices decreases with distance from the inlets (sedimentation of progressively finer fractions). The exceptions were short periods in which a large amount of waste accumulated on the bar screen slowed down the flow from the very beginning.

The research results showed a strong influence of grit chamber 1 on the quality of sludge in the settler. At measuring point 1c, the average concentrations of Zn, Cu and Pb are higher by 51.7, 53.3 and 57.0%, respectively, than at point 2c, adjacent to grit chamber 2. A significant decrease in sediment pollution occurs during the transition from the settler to the retention pond—ranging from about 40% (Zn and Cu) to 52% (Pb) on average (Table 2). The lower content of metals in the reservoir may indicate their good binding to the solid particles, which in the vast majority are retained in the grit chambers and the settler. This is facilitated by a pH close to neutral. With the exception of 2009 (an increase in pollution), the concentration of metals in sediments of the retention pond (points 3a and 3b) was comparable in subsequent measurement periods. For other devices of the sewage treatment plant, high temporal variability of pollutant accumulation was reported. For example, the amount of Pb retained in grit chambers 1 and 2 decreased with time. The opposite trend was noticed in grit chamber 2 for concentrations of Zn and Cu.

In comparison to the data provided by other authors, the level of pollution of the sludge from the studied wastewater treatment plant is not high. Due to the difficulty in finding information in the literature about a similar facility (a large urban system consists of typically technical and semi-natural units), in Table 3, the obtained results were collated with the size of the accumulation of metals in pond systems for stormwater management. Comparing the share of particular trace elements, similar tendencies can be observed. The following order of concentration dominates: Zn > Pb > Cu; less commonly: Zn > Cu > Pb. The highest pollution was observed in the case of rainwater run-offs from roads with heavy traffic and industrial areas (Allinson et al. 2015; El-Mufleh et al. 2014).

**Table 3:** Comparison of average concentrations of metals in sludge of the treatment plant in Puławy (own studies) with average accumulation in exemplary rainwater reservoirs (literature data)

Variables	Treatment plant in Puławy				Literature data			
	Grit chamber 1	Grit chamber 2	Settler	Retention pond	Wetlands, retention ponds^a^	Infiltration basin^b^	Detention pond^c^	Drainage system ponds^d^
Zn (mg kg^−1^)	248.4	112.0	156.3	94.2	495.0	1586.2	189.0	89.6
Cu (mg kg^−1^)	19.7	8.6	11.0	6.7	51.0	244.8	51.3	18.4
Pb (mg kg^−1^)	30.4	14.2	21.0	10.1	78.0	338.8	34.0	23.1

^a^Allinson et al. (2015)

^b^El-Mufleh et al. (2014)

^c^Färm (2002)

^d^Heal et al. (2006)

The reference data indicates that the suspension in rainwater may also be related to the presence of other kinds of pollutants. For example, a relationship between the concentrations of suspensions and the level of chemical oxygen demand (COD) and the concentration of nutrients and turbidity was found. The size of the particles forming a suspension is very important. Nutrients are usually transported with finer and slower settled fractions (Vaze and Chiew 2004; Wakida et al. 2014). The suspension studies deposited on roads showed similar correlations for heavy metals (Gunawardana et al. 2014; Zhao et al. 2010). In the context of this information, the separation of zones with different hydraulic conditions in the stormwater treatment plant in Puławy seems to be the right move, because of the ability to retain the different fractions. The amount and fractional composition of dust from urbanised areas are closely connected with the type of drained surfaces. Shen et al. (2016) demonstrated that medium and coarse grains of suspension are characteristic to road pollution, while the smallest particles lie usually on roofs. Petrucci et al. (2014) proposed a methodology of analysis of sources of pollution in rainwater run-offs. According to them, roofing accessories should be included as important suppliers of Zn and Pb, while the phenomena connected with human activity (traffic and heating) are associated with the emission of Cu.

Products of tyre and brake disc abrasion and exhaust gases may be the direct sources of Zn, Cu and Pb in rainwater run-offs from urban transport routes. Zn and Pb may also be mentioned among products of road surfaces wearing down and construction materials. Trace metals, in some cases, are also released from painted surfaces, plastics, rubber products, metal alloys, pesticides, slag and ash. A significant anthropogenic source of Zn, Cu and Pb in the environment is also the burning of fossil fuels and waste (Kabata-Pendias and Mukherjee 2007; Królikowski et al. 2006; Pacyna and Pacyna 2001; Petrucci et al. 2014). These sources and processes are commonly present in the catchments presented in the article.

Operating treatments performed in the studied object comprise periodic removal of sludge retained in the grit chambers and the settler. It has been estimated that with a 1-cm layer of sediments, taking into account the dry mass and average contents of metals, 24.2 kg of Zn, 1.7 kg of Cu and 3.2 kg of Pb are discharged from the stormwater treatment plant. For safety, sediments should undergo remediation processes. Biological methods are increasingly popular, among which phytoremediation and phytoextraction can be distinguished (Bert et al. 2009). Some species of vascular plants have a high ability for uptake and accumulation of contaminants, including heavy metals (Hegazy et al. 2011; Kara 2005; Kumari and Tripathi 2015). At the same time, particular attention should be paid to the need for selecting appropriate plants in relation to the prevailing habitat conditions and the type of eliminated components. In the analysed object, thanks to the large availability of the land, there is the possibility of separating the remediation plots planted with vascular plants. The need to distribute the hydrous (raw) sediments on this type of surface and the duration of the growing season in the temperate climate zone require modification of operational activities—postponement of the removal of sludge from the autumn to the turn of winter and spring. Despite the high diversity of the degree of pollution, sediments from all stages of purification should be deposited on a remediation plot (the precautionary principle). Their good mixing will result in partial alignment of composition (dilution of pollutants).

Due to the high durability and capacity to accumulate trace metals in ecosystems, constant monitoring of the quality of rainwater sludge is required. According to the literature data, excessive concentrations of Zn, Cu and Pb are harmful to living organisms. In the case of plants, symptoms of toxic action may be inhibition of photosynthesis, decreased yield and dieback (Zn, Cu and Pb), dysfunctional root development and DNA damage (Cu and Pb) and impaired water economy (Pb) (Kabata-Pendias and Mukherjee 2007; Wilk and Gworek 2009). The effects of high doses of analysed metals on animals and humans can cause gastrointestinal disorders, osteoporosis, lymph node dysfunction (Zn), dysfunction of DNA and certain enzymes, cell damage, allergies, hypertension, diabetes, depression (Cu and Pb), nervous system damage, renal failure, anaemia, cancers, infertility, decreased immunity and anorexia (Pb) (Lokeshappa et al. 2012; Seńczuk 2002).

## Summary

A by-product of the functioning of the stormwater treatment plant includes sludge. Its periodic removal is one of the basic operational activities for maintaining the appropriate hydraulic conditions in the facility. Insufficient research and modest knowledge of sludge from a storm sewage system lead to a lack of precise indications on the methods for dealing with it. In many countries, municipal services treat it as an inert material, not taking the appropriate precautions (e.g. deposition in unsecured places). Meanwhile, studies have shown that sediments are a convenient place for the deposition of chemical pollutants, including heavy metals. Putting them into the environment in a disorganised manner may result in the release and uncontrolled migration of harmful components. According to the authors of this article, the sediments must be seen as a potentially hazardous waste in chemical terms. It is necessary to use appropriate methods of quality control and precisely assess the level of pollution; in addition to the cognitive aspect, it is also important to establish generally applicable methods for removal and disposal (currently, in Poland, there are no regulations on sludge from rainwater sewage).

The content of heavy metals in the sediments of the analysed wastewater treatment plant shows a significant variation, which confirms the initial research hypothesis. This is due to the large size of the object and the presence of many devices with different hydraulic conditions (sequential sedimentation of suspended solids). The decisive factor for the significant variation of sediment pollution may be the method and intensity of use of the drained surfaces (a tributary of the two zones of the town). The highest concentrations of Zn, Cu and Pb were recorded in the sediments accumulated from the side of the interceptor transporting rainwater sewage from the main traffic route of the town with heavy traffic (grit chamber 1, the northern part of the settler). The smallest pollution characterised the sludge from the retention pond (the last element of the rainwater treatment plant), from which the treated sewage is discharged into the receiver. In the sediments of such a large object as municipal stormwater treatment plants, the differences in the content of heavy metals in a single date may reach 467%—Zn, 432%—Cu and 872%—Pb (comparison of positions with extreme values). Zinc (Zn) has the largest share in the pool of surveyed pollutants and, depending on the device, totals, on average, from 83% (the grit chambers and the settler) to 85% (the retention pond), while copper (Cu) has the lowest—from 6% (grit chamber 2, the settler and the retention pond) to 7% (grit chamber 1).

For safety reasons, the sludge from storm sewage should be stabilised, e.g. in the processes of bioremediation (natural renewal). To do this, the ability of higher plants to accumulate selected components of pollutants and the removal of them together with biomass (phytoremediation) can be used. The appropriate assessment of habitat conditions, the selection of species and the schedule of treatments over time are very important here.
